# The Influence of PRO-SELF Cancer Pain Control Programme on Patients’ Self-Management Ability

**DOI:** 10.5334/ijic.7543

**Published:** 2023-10-30

**Authors:** Xin Yin, Zi-Jing Chu, Yuan-Yuan Ni, Hong-Wei Li, Hong-Yan Li

**Affiliations:** 1 Department of Nursing, The First Hospital of Jilin University, Changchun 130021, China; 2 Department of Hepatology, The First Hospital of Jilin University, Changchun 130021, China; 3 Department of Infirmary, WuXi Institute of Technology, WuXi, 214000, China; 4 Department of Oncology, The First Hospital of Jilin University, Changchun 130021, China; 5 Dean Office, The First Hospital of Jilin University, No. 1 of Xinmin Street, Chaoyang District, Changchun 130021, China

**Keywords:** PRO-SELF, cancer pain, self-management, anxiety and depression, nursing

## Abstract

**Objective:**

Recently, cancer patients have challenges with self-management. This study aims to improve symptoms of chronic pain, and anxiety and depression associated with cancer by PRO-SELF nursing intervention.

**Methods:**

Sixty-four patients were randomly assigned to an intervention and a control group from Jan 2016 to Dec 2019, 34 usable cases in the intervention group and 30 cases in the control group were collected. The control group received a routine cancer pain nursing intervention, whereas the intervention group received a PRO-SELF based multidisciplinary collaborative cancer pain nursing intervention. After three months of intervention, the Numeric Rating Scale (NRS), Medication Compliance Questionnaire (MCQ), Social Support Rating Scale (SSRS), Self-rating Anxiety Scale (SAS) and Self-Rating Depression Scale (SDS), Quality of Life Scale (FACT-G Chinese version) and Chronic Pain Self-efficacy Scale were resent to compare the differences in the observation indicators included evaluation of patients’ social support degree, anxiety and depression score, quality of life scores and self-efficacy scores between two groups. The t-test and rank-sum test were used for statistic analysis.

**Results:**

No significant differences were found between groups for pain and medication compliance (P > 0.05). However, significant differences were found between groups in social support, life quality, chronic pain self-efficacy, and self-rating anxiety and depression index scores (P < 0.001). The intervention group report more social support, pain self-efficacy and less anxiety and depression (P < 0.001).

**Conclusion:**

The PRO-SELF pain symptoms in patients with a cancer pain management programme improved degree of social support, life quality, self-efficacy, anxiety, depression which is worthy of clinical application.

## Introduction

Pain is the most common clinical symptom of cancer patients, which seriously affects their quality of life [1]. Along with the prolongation of the tumour survival time, the discharged patients receiving continuous, complete treatment and nursing services directly affect self-care behaviour and quality of life [1]. With the establishment of the biopsychosocial medical model that proposed by George Engel in 1977 [2], there is an urgent need to establish a continuous nursing service extending from hospital to community and family to solve the problems, including the lack of medical and health information, nursing services interruption and the difficulties involved in meeting patients’ demand after discharge [3]. Self-management referred to the individual to maintain and improve their health by their actions, monitor and manage their symptoms in order to reduce the impact of disease on one’s social functioning, emotions and relationships.

The PRO-SELF cancer pain symptom self-management programme was created and developed by Dodd [4] in 2000. The project is based on Orem’s self-care theory. It mainly provides information, techniques and support to adult patients who receive cancer treatment to enable them to participate more effectively and consistently in the created self-symptom management programme to reduce the serious symptoms caused by the disease. A clinical study in the Netherlands indicated that self-management support was expected to lead to better pain control and better quality of life compared to usual care [5]. Elmokhallalati proposed that clinicians should integrate evidence-based activities into routine clinical practice to support the intervention of cancer pain self-management [6]. However, few studies have applied self-management to cancer pain in China, especially for cancer pain patients after discharge.

This prospective study takes the pain PRO-SELF strategy as a guide on the continuous nursing intervention for discharged cancer patients. This study intended to evaluate the impact of the pain PRO-SELF strategy on the self-management ability of patients with cancer pain. It subsequently enables the development of an effective pain self-management programme and provides a scientific basis for the effective improvement in patients’ self-management ability.

## Materials and methods

### Subjects

This study aims to investigate the influence of PRO-SELF cancer pain symptom control programme on patients’ self-management ability. Excluding patients who did not meet the inclusion criteria, and patients who did not complete the study due to migration, self-withdrawal, death and other reasons, 64 patients were finally included in this study from Jan 2016 to Dec 2019. The 64 patients were divided into an intervention group (34 cases) and a control group (30 cases).

### Study method

This was a prospective case control study. Patients were recruitment from the outpatient department of cancer centre in our hospital from Jan 2016 to Dec 2019. At the time of enrollment, patients completed a demographic questionnaire, the Karnofsky performance score [7] and for 3 days before the first study visit, patients rated their level of pain intensity on a daily basis. Participants were randomly divided into the intervention group and the control group according to the random number table. The allocation sequence was generated by computer. No blinding was designed in this study. The sample size was determined by the PASS software. The data collection methods was conducted by CRF forms. Data were collected in the form of CRF forms according to the medical history of patient and the relevant scales filled out.

This study was conducted in accordance with the Declaration of Helsinki and approved by the ethics committee of our hospital. All participants had signed the informed consent. The primary endpoint was the patients pain control, the secondary endpoints were medication compliance, social support, anxiety and depression, quality of life and self-efficacy.

### Inclusion and exclusion criteria

The inclusion criteria included (1) older than 18 years old; (2) had cancer with pain diagnosed by histopathology; (3) had Karnofsky Performance Status (KPS) score of ≥50; (4) had average pain intensity scores of ≥2.5; (5) had been aware of their illness and its diagnosis; (6) expected to survive for more than 3 months; (7) have had no language barrier and have had no history of mental illness; (8) be able to read or complete the questionnaire with or without the researchers’ help, understand the study and volunteer to participate in the experiment.

The exclusion criteria included (1) bone metastases; (2) taking additional painkillers outside of the prescription; (3) suffer from language communication disorders; (4) unclear consciousness or mental disorders.

### Creation of a multidisciplinary collaborative cancer pain intervention team

In our PRO-SELF cancer pain symptom control programme, we formed a multidisciplinary collaborative cancer pain intervention team. The team composed of tumour doctors, pain doctors, psychologists, counsellors, pharmacists, cancer pain nurses and social workers. The pain specialist nurses led a multidisciplinary team. They tried to apply their nursing professional knowledge in the clinical area and strengthen communication and cooperation with the multidisciplinary staff. They also participated in the personalisation and optimisation of the patients’ diagnosis and treatment plans to ensure that all measures were effectively implemented and tracked. They also ensured that the treatments’ effects were fed back to enable them to provide the physicians with complete and accurate clinical information of their patients to ensure successful clinical diagnosis and treatment [3].

The pain specialist nurses contacted with participants or their family caregivers once a week (Every Tuesday or Wednesday from 2:00 to 4:00 p.m) by office phone in Oncology Department. The control group was given a routine cancer pain nursing intervention, while the test group was given a multidisciplinary collaborative cancer pain nursing intervention based on PRO-SELF. During the first visit, we provided patients with written instructions on pain and side effect management, teaching them how to use weekly medication boxes and communicating with doctors about unresolved pain and the need to change pain medication prescriptions. At weeks 4, 8, and 10, the pain specialist nurse contacted the intervention group patients by phone and strengthened the education content of the PRO-SELF, guiding patients on how to modify their pain management plan or how to contact their doctor to improve pain outcomes. PRO-SELF nurses focus on guiding pain management in the following areas: how to evaluate pain and patient response to analgesics; how to improve pain by changing the frequency and frequency of painkillers taken without exceeding the prescribed dosage; how to prevent and manage side effects related to analgesics; and how to communicate with clinical doctors about unresolved pain and the need to change their pain relief prescriptions.

### Creation of a multidisciplinary nursing intervention programme for cancer pain based on PRO-SELF

Based on the PRO-SELF pain self-management project, this study combined the evidence-based needs of the nursing patients and the multidisciplinary pain control mode to set up a nurse-led multidisciplinary PRO-SELF cancer pain control scheme. To improve the patients’ self-management ability, the intervention was designed in three parts consisting of information, support and skills in six time nodes, including the 2, 4, 6, 8, 10, 12 week after enrollment. The investigators recruited the patients provided the intervention, the content included evaluation of patients’ social support degree, anxiety and depression score, quality of life scores and self-efficacy scores after three months of intervention.

### Organising standardised training for nurses

Three pain specialist nurses were trained on the PRO-SELF-based multidisciplinary nursing intervention programme for cancer pain and its specific operation methods. The training included the composition and flow of the PRO-SELF pain nursing intervention programme, the working mode and method of the multidisciplinary team, the operation and implementation of specific interventions, such as the contents and methods of pain assessment or tools of pain. In total, the programme consisted of 10 hours to ensure the implementation of the standardised nursing intervention.

### Application and effect evaluation of the multidisciplinary collaborative cancer pain nursing intervention programme based on PRO-SELF

#### Numeric rating scale (NRS)

NRS was used to assessed pain, allowing for pain scores between 0 (no pain) and 10 (worst pain) [8]. Pain was defined as mild for NRS values 1–3, moderate for NRS values 4–6, and severe for NRS values ≥7. Patient was eligible for the study when their NRS values ≥1.

#### Medication compliance questionnaire (MCQ)

Chinese version of Morisky, LL Gen drug compliance Scale was used (MMAS 1:8) [9]. This scale is a universal drug dependence scale, with 8 items in total. The full score of the scale is 8 points, and the score <6 is low in dependency: score 6~7 is medium, and score 8 is high in compliance.

#### Social Support Rating Scale (SSRS)

Social Support Rating Scale was developed by Xiao Shuiyuan in 1986 [10]. The scale contains 10 items and can be divided into objective support, subjective support and support degree dimensions respectively. The Scale scores ranged form 0–66 and values of the score <23 for low level, 23~44 for moderate level, and 45~66 for high level. The consistency of each item is between 0.89–0.94, indicating that the questionnaire has a good internal consistency.

#### Self-rating anxiety and depression scale

Self-rating Anxiety Scale (SAS) and Self-Rating Depression Scale (SDS) were developed by W.K. Zung and widely used in China [11]. The SAS and SDS contain 20 items that reflect subjective feelings of anxiety and depress. In the Chinese version, the value of SAS^3^50 indicates anxiety and the value of SDS^3^53 indicates depress. Their reliability and validity were pretty good in Chinese patients with cancer pain.

#### Quality of life scale (FACT-G Chinese version, v4.0)

FACT-G was developed by American scholar Cella and translated by Wang Chonghua [12]. Measuring quality of life of Chinese cancer patients: a validation of the Chinese version of the Functional Assessme.

### Data collection

For the time of the patients’ admission, we provided the following five scales for the intervention and control groups: NRS, MCQ, SSRS, SAS and SDS FACT-G and Chronic pain self-efficacy scale for the first evaluation [10], these scales were given for pain control, medication compliance, social support, anxiety and depression, quality of life and self-efficacy.

After three months, the six scales were resent to compare the differences in results between the test and control groups in terms of pain control, medication compliance, social support, anxiety and depression, quality of life and self-efficacy. These results were then used to appraise the impact of the multidisciplinary collaborative cancer pain nursing intervention on patients’ self-management ability.

### Statistical analysis

Excel was used to create a database, and the SPSS 17.0 software was employed for statistical analysis. Data conforming to normal distribution were checked by the t-test and variance analysis. Data conforming to non-normal distribution were checked by the rank-sum test. Countable data were checked by the chi-square test.

## Results

### Comparison of the general situation between the two groups of patients

No significant difference was observed between the two groups in terms of gender, marital status, educational level, occupation and monthly income of the family (P > 0.05) (see Table 1).

**Table 1:** The general comparison between two groups of patients.


ITEMS		INTERVENTION GROUP (34)	CONTROL GROUP (30)	STATISTIC VALUE	*P*

Gender	Male	18	20	0.138	0.265

	Female	16	10		

Age		55.12 ± 10.73	57.80 ± 8.47	–1.100	0.276

Marriage	Married	26	28	2.289	0.119

	Single	2	2		

	Lose the spouse	2	0		

	Divorced	4	0		

Education	Primary school	2	6	0.349	0.064

	Middle school	16	14		

	High school	8	6		

	Vocational school	2	4		

	Bachelor or higher	6	0		

Career	Worker	10	10	0.190	0.693

	Farmer	6	4		

	Intellectual	10	4		

	Individual business	2	3		

	Others	6	5		

Family income	1000–3000 yuan	26	22	0.036	0.772

	3000–6000 yuan	8	8		


### Comparative scores of social support degree of patients before and after the intervention

After a three months’ intervention, no significant difference was seen between the two groups of patients in terms of pain and medication compliance scores. After the intervention, significant differences were observed in the total score of social support, objective support and subjective support between the two groups, and the scores of the intervention group were higher than those of the control group (P < 0.001), as shown in Table 2.

**Table 2:** Comparison analysis of SSRS scores of two groups after a three months’ intervention.


GROUP	OBJECTIVE SUPPORT	SUBJECTIVE SUPPORT	SUPPORT DEGREE	TOTAL

Intervention group	12.67 ± 6.87	18.61 ± 5.94	6.56 ± 2.22	37.83 ± 9.92

Control group	8.39 ± 1.89	15.71 ± 4.14	6.13 ± 2.13	29.58 ± 6.97

t value	3.656	2.587	0.945	4.401

*P*	0.001	0.012	0.348	<0.001


### Comparison of anxiety and depression scores between the two groups before and after the intervention

The anxiety and the index of depression severity scores of the two groups after the three months of intervention were significantly different, and the scores of the intervention group were lower than those of the control group (P < 0.001), as shown in Table 3.

**Table 3:** Comparison analysis of anxiety score, depression index, quality of life and self-efficacy scores of two groups after a three months’ intervention.


GROUP	INTERVENTION GROUP	CONTROL GROUP	T VALUE	P

Score of anxiety	47.43 ± 5.30	53.92 ± 3.31	–6.605	<0.001

Score of Depression	0.50 ± 0.087	0.58 ± 0.055	–4.422	<0.001

Physiological status	16.00 ± 4.50	12.20 ± 3.94	4.34	<0.001

Social/family status	15.28 ± 4.80	16.33 ± 4.62	–1.067	0.289

Emotional status	12.28 ± 2.55	11.10 ± 1.16	2.836	0.007

Functional status	9.72 ± 2.48	9.80 ± 2.53	–0.147	0.884

Chronic pain	15.06 ± 3.68	10.13 ± 3.61	6.426	<0.001

Somatic function	22.78 ± 8.74	15.07 ± 4.25	4.953	<0.001

Symptom coping	23.67 ± 4.20	16.27 ± 4.06	8.541	<0.001


### Comparison of quality of life scores between the two groups before and after the intervention

After the three months of intervention, significant differences were seen in the total scores of life quality, as well as physiological and emotional statuses between the two groups, and the scores of the intervention group were higher than those of the control group. However, no significant difference was noticed in the social/family and functional statuses (P = 0.037), as shown in Table 3.

### Comparison of self-efficacy scores between the two groups before and after the intervention

Significant differences were observed between the two groups of patients after the intervention in three dimensions: (a) self-efficacy and the overall score of chronic pain self-efficacy, (b) physical function self-efficacy and (c) symptoms coping self-efficacy. And the scores of the intervention group were higher than those of the control group (P < 0.001) (see Table 3).

## Discussion

The results of this study illustrated that the scores of social support, quality of life, self-rating anxiety scale, self-rating depression scale and chronic pain self-efficacy in the patients receving the PRO-SELF cancer symptoms self-management project were significantly better than those in the control group, suggesting that the pain PRO-SELF strategy can improve social support, comprehensive quality of life, self-efficacy, anxiety and depression index of patients with cancer pain.

Patients’ self-management included hospital intervention and continuous nursing intervention after discharge. Hospital interventionin included evaluation of the symptoms of patients, providing personalized patient education content in view of the actual situation, pain treatment, relief skills strategy, distribution of pain knowledge manual, emotional support. Continuous nursing intervention after discharge included telephone follow-up or regular home visits, providing emotional support to the patient, medication guidance and cancer pain relief strategies at home.

The PRO-SELF cancer pain symptom self-management programme was created and developed by Dodd [4] in 2000. This programme consisted of three parts [13]: ①information on the patients’ treatment and self-management, ②self-care exercise to help patients acquire sufficient skills to manage their symptoms and ③support, interaction, care, nursing, coaching to enable listening to patients and actively encourage patients to self-care and provide incentives for treatment experiences. Studies [14,15] have shown that the PRO-SELF cancer symptoms self-management project can make an important contribution to the improvement of the self-management ability of cancer patients and a reduction in the incidence of disease. They have also shown that the project has positive potentials for improving the self-management ability of cancer patients [16,17].

The results illustrated no significant difference in pain and medication compliance scores among patients in the two groups after the three months’ intervention, which is attributed to the defect of the degree of pain, medication compliance and disease, among others. A significant difference was seen in social support, life quality, self-rating anxiety scale, self-rating depression index and chronic pain self-efficacy scores in the two groups of patients before and after the intervention. This finding is consistent with Long Haiyan’s [18] and He Haiyan’s [19] results. They assert that comfort care, health education, skill learning, exercise, emotional support and other interventions can improve the psychological status, quality of life and pain self-efficacy of cancer patients. The results illustrate the suitability of the pain PRO-SELF strategy for clinics.

### Implications for Nursing

Multidisciplinary collaboration of pro-self provides personalized support for patients, and ultimately improves patients’ self-management ability by improving patients’ self-management cognition level [20,21,22,23,24]. The program provided individualized evaluations and support, which may have made it easier for participants to accept the information and learn skills. This study also suggested the importance of continuing to provide multidisciplinary and personalized support to patients after discharge. In future studies, scholars should pay special attention to provide patients with information and technical support in various forms and easy to accept through the internet after discharge, so as to ensure the continuous improvement and maintenance of self-management ability [25,26,27].

Limitations. There were several limitations in this study. Firstly, this trial had no blinding. Secondly, this study was only single-center trial and the sample size was limited. Thirdly, the clinical follow-up was short and it was necessary to observe the clinical long term efficacy. Further studies with multicentre randomized controlled trials and larger populations of cancer patients are necessary to evaluate the effect of this PRO-SELF pain symptom control programme.

## Conclusion

The PRO-SELF pain symptoms in patients with a cancer pain management programme improved degree of social support, life quality, self-efficacy, anxiety, depression and other negative emotions which is worthy of clinical application.

## Data Accessibility Statement

The data underlying this article will be shared at reasonable request to the corresponding author.

## References

[1] Chen H, Yao ZJ, Cai MH, Zhu HP. Qualitative research on nursing needs of patients with cancer pain. Journal of Bengbu Medical College. 2015; 12: 1762–1764.

[2] Park EW, Engel, GL. Biopsychosocial medical model. Journal of the Korean Academy of Family Medicine. 2000; 21(10): 1223–1226.

[3] Zhang PW, Wang W, Liu S, Zhao JJ, Zhou LJ. Construction and application of nursing scheme in cancer pain management. Nursing research. 2012; 5: 417–418.

[4] Dodd MJ, Miaskowski C. The PRO-SELF program: A self-care intervention program for patients receiving cancer treatment. Seminars in Oncology Nursing. 2000; 16(4): 300–308. DOI: 10.1053/sonu.2000.16586 11109273

[5] Hochstenbach LM, Courtens AM, Zwakhalen SM, van Kleef M, de Witte LP. Self-management support intervention to control cancer pain in the outpatient setting: a randomized controlled trial study protocol. BMC Cancer. 2015; 15: 416. DOI: 10.1186/s12885-015-1428-1 25986294 PMC4451734

[6] ElMokhallalati Y, Mulvey MR, Bennett MI. Interventions to support self-management in cancer pain. Pain Rep. 2018; 3(6): e690. DOI: 10.1097/PR9.0000000000000690 30706035 PMC6344141

[7] Karnofsky D. Performance scale. In: Kennealey GT, Mitchell MS (eds.) Factors That Influence the Therapeutic Response in Cancer. New York, NY: Plenum Press; 1977.

[8] Gagnon CM, Scholten P, Atchison J, Jabakhanji R, Wakaizumi K, Baliki M. Structural MRI Analysis of Chronic Pain Patients Following Interdisciplinary Treatment Shows Changes in Brain Volume and Opiate-Dependent Reorganization of the Amygdala and Hippocampus. Pain Med. 2020; 21(11): 2765–2776. DOI: 10.1093/pm/pnaa129 32488262 PMC8463093

[9] Xiao S, Zhao C, Sun J, Dou Y, Teng M. Effect of high-quality nursing on negative psychological moods and quality of life of elderly patients with hypertension. Am J Transl Res. 2021; 13(4): 3710–3716. 34017555 PMC8129218

[10] Wu C, Li S, Cheng F, Zhang L, Du Y, He S, et al. Self-Identity and Career Success of Nurses in Infectious Disease Department: The Chain-Mediating Effects of Cognitive Emotion Regulation and Social Support. Front Psychol. 2020; 11: 563558. DOI: 10.3389/fpsyg.2020.563558 33329191 PMC7729080

[11] Gu X, Song LJ, Li LX, Liu T, Zhang MM, Li Z, et al. Fusobacterium nucleatum Causes Microbial Dysbiosis and Exacerbates Visceral Hypersensitivity in a Colonization-Independent Manner. Front Microbiol. 2020; 11: 1281. DOI: 10.3389/fmicb.2020.01281 32733392 PMC7358639

[12] Meier-Girard D, Ribi K, Gerstenberg G, Ruhstaller T, Wolf U. Eurythmy therapy versus slow movement fitness in the treatment of fatigue in metastatic breast cancer patients: study protocol for a randomized controlled trial. Trials. 2020; 21(1): 612. DOI: 10.1186/s13063-020-04542-5 32631427 PMC7336433

[13] Belschak FD, Den Hartog DN. Pro-self, prosocial, and pro-organizational foci of proactive behaviour: Differential antecedents and consequences. Journal of Occupational and Organizational Psychology. 2010; 83(2): 475–498. DOI: 10.1348/096317909X439208

[14] Valeberg BT, Kolstad E, Småstuen MC, Miaskowski C, Rustøen T. The PRO-SELF Pain Control Program Improves Family Caregivers’ Knowledge of Cancer Pain Management. Cancer Nursing. 2013; 36(6): 429–435. DOI: 10.1097/NCC.0b013e3182747bcf 23154516

[15] Sutters K A, Savedra M C, Miaskowski C. The pediatric PRO-SELF(c): pain control program: an effective educational program for parents caring for children at home following tonsillectomy. J Spec Pediatr Nurs. 2011; 16(4): 280–294. DOI: 10.1111/j.1744-6155.2011.00299.x 21951354 PMC4523122

[16] Zhao JJ, Cui J. The role of nurses in pain management. Chinese Journal of nursing. 2009; 4: 383–384.

[17] Rustøen T, Valeberg BT, Kolstad E, Wist E, Paul S, Miaskowski C. The Pro-Self© Pain Control Program Improves Patients’ Knowledge of Cancer Pain Management. Journal of Pain and Symptom Management. 2012; 44(3): 321–330. DOI: 10.1016/j.jpainsymman.2011.09.015 22704056

[18] Long HB, Wang QL, Zhao H, Liang L. Effect of comfortable nursing intervention on improving psychological status and quality of life of patients with cancer pain. International Journal of Psychiatry. 2015; 42(6): 111–114.

[19] He HY. Influencing factors and nursing intervention of cancer pain self efficacy. Third Military Medical University; 2008.

[20] Yates P, Edwards HE, Nash RE, Aranda S, Purdie D, Najman J, et al. A randomized controlled trial of a nurse-administered educational intervention for improving cancer pain management in ambulatory settings. Patient Education & Counseling. 2004; 53(2): 227–237. DOI: 10.1016/S0738-3991(03)00165-4 15140463

[21] Buck R, Morley S. A daily process design study of attentional pain control strategies in the self-management of cancer pain. European Journal of Pain. 2012; 10(5): 385–385. DOI: 10.1016/j.ejpain.2005.04.001 15946872

[22] Lovell MR, Luckett T, Boyle FM, Phillips J, Agar M, Davidson PM. Patient education, coaching, and self-management for cancer pain. Journal of Clinical Oncology. 2014; 32(16): 1712–20. DOI: 10.1200/JCO.2013.52.4850 24799486

[23] Chatwin J, Closs J, Bennett M. Pain in older people with cancer: attitudes and self-management strategies. European Journal of Cancer Care. 2010; 18(2): 124–130. DOI: 10.1111/j.1365-2354.2007.00885.x 19267727

[24] You E. Nontraditional and Home-Based Self-management Interventions in Cancer Patients With Pain: A Mixed-Method Systematic Review. Holistic nursing practice. 2020; 34(3): 138–149. DOI: 10.1097/HNP.0000000000000380 32282489

[25] Silva EH, Lawler S, Langbecker D. The effectiveness of mHealth for self-management in improving pain, psychological distress, fatigue, and sleep in cancer survivors: a systematic review. Journal of cancer survivorship: research and practice. 2019; 13(1): 97–107. DOI: 10.1007/s11764-018-0730-8 30635865

[26] Eaton LH, Hulett JM. Mind-Body Interventions in the Management of Chronic Cancer Pain. Seminars in Oncology Nursing. 2019; 35(3): 241–252. DOI: 10.1016/j.soncn.2019.04.005 31053397

[27] Hung CT, Chen YJ, Chan JC, Fang YY, Li IF, Shih HH, et al. Psychological distress, social support, self-management ability and utilization of social resources for female patients with cancer in Oncology Outpatient Settings in Taiwan. Supportive Care Cancer. 2019; 28(2): 3323–3330. DOI: 10.1007/s00520-019-05143-y 31758322

